# Cystinuria: urine sediment as a diagnostic test

**DOI:** 10.1515/almed-2020-0026

**Published:** 2020-05-04

**Authors:** María Pierna, Mohamed Abdelgabar, Raquel Fernández-Rivas, Miguel Fernández-Burriel

**Affiliations:** Department of Clinical Analyses, Hospital de Mérida, Mérida, Badajoz, Spain; Emergency Department, Hospital de Mérida, Mérida, Badajoz, Spain; Department of Clinical Analyses, Hospital de Mérida, Polígono Nueva Ciudad s/n, 06800, Mérida, Badajoz, Spain

**Keywords:** cystinuria: cytine crystals, renal calculi, urine sediment

## Abstract

**Objectives:**

To demonstrate the importance of carrying out the urinary sediment study with the correct interpretation and crystals typification as a clinical laboratory diagnostic tool, as well as the elaboration of protocols that determine the need to realize this type of microscopic urinary sediment examination routinely.

**Case presentation:**

Elderly male patient with no personal or family history of interest that presented with left iliac fossa fixed and non-irradiated pain lasting three days. This is the first time that he suffered pain episodes of this type. The urine analysis reveals proteinuria, hematuria and the sediment shows abundant flat and hexagonal crystals, typical of cystine. Amino acid analysis confirms the finding, showing high dibasic amino acids and cystine concentrations.

**Conclusions:**

The study of the urinary sediment by the clinical laboratory reveals the presence of a case of cystinuria due to the appearance of their pathognomonic crystals at an advanced age and without a previous history. The case reported in this paper is of interest for clinical laboratory practice, as it demonstrates the utility of urine sediment examination in the diagnosis of a genetic disease that manifests as a simple renal colic.

## Introduction

Cystinuria belongs to the group of hereditary aminoacidurias. This condition has an overall prevalence of 1:7,000 [1] and is characterized by a defect in transepithelial transport of the amino acid cystine and the dibasic amino acids ornithine, lysine, and arginine. This defect causes and impaired intestinal and renal absorption of amino acids. The amino acids filtrated in the glomerulus are deficiently reabsorbed in the proximal renal tubule, which results in the accumulation of amino acids in urine. As urine becomes more concentrated, the excess cystine precipitates into crystals, mostly in acidic urine, which can coalesce into cystine calculi [2, 3].

Although cystinuria is the most common of hereditary aminoacidurias, its clinical manifestation (e. g. urinary tract cystine calculi) is a rare type of nephrolithiasis [4]. This disease is associated with mutations in two genes: *SLC3A1*, which encodes the heavy subunit of the cystine and basic amino acid transporter (b(0,+)) rBAT (related b^or,+^ aminoacid transporter); and *SLC7A9*, which encodes the functional unit or light subunit of that transporter b(0,+)AT [2, 3].

The most common presenting symptoms are those typical of urinary tract calculi, namely: microscopic and macroscopic hematuria, renal colic with or without excretion of calculi, and lumbar pain. These symptoms have high recurrence and may occur concomitantly with urine infection, urinary tract obstruction and, occasionally, renal failure [5]. Cystine calculi are yellowish with a waxy appearance; although they are radiopaque because of their high density and the presence of sulfur molecules in their composition, abdominal radiological examination has low sensitivity and only detects 50% of cases [6].

Urine sediment examination is useful for the classification of lithiasis based on the characteristics of crystals and is conclusive for a clinical diagnosis of cystinuria. Cystine crystals are pathognomonic of cystinuria and show a flat hexagonal structure, which thickness may reach 1 mm in phases of activity. Thus, this paper presents a case of interest for clinical laboratory practice, as it demonstrates the utility of urine sediment examination as a diagnostic tool in the evaluation of a genetic disease, which first episode generally occurs at an adult age and can remain unnoticed in a first evaluation of the case.

## Case presentation

We report the case of a 49 year-old patient not taking regular medication without any personal or family history of interest who presented to the emergency department (ED) of his hospital of reference, where he was administered intramuscular metamizole and metoclopramide for pain management. After 48 h, the patient came back to the ED of our hospital with a 3-day history of persistent pain in the left iliac fossa without irradiation, with negative bilateral fist percussion of the kidney. The patient reported that this was the first episode he suffered and did not describe dysuria or urinary urgency, although he complained of frequent urination.

**Figure 1:**
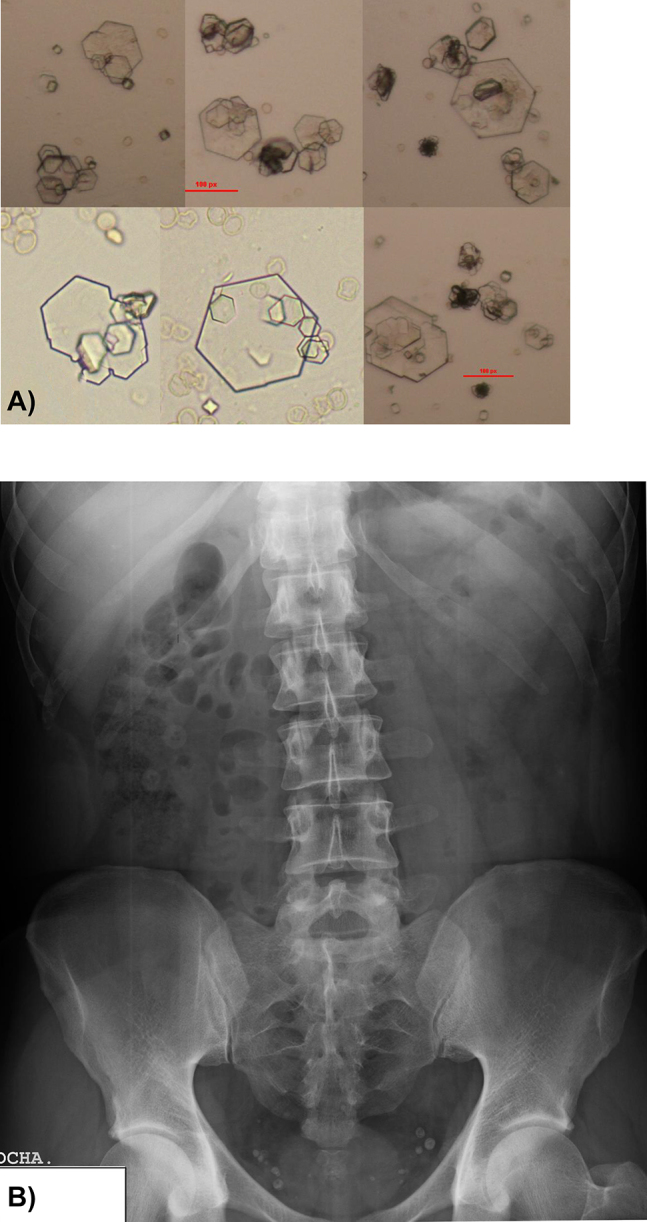
(A) Urine sediment on admission (400×). Small, flat, hexagonal-shaped crystals (10–20 µm) with different levels of twinning. Presence of medium-sized and large twinnings arranged in the layers or ladder typical of this type of crystals. (B) Simple abdominal X-ray showing the presence of several phleboliths unrelated to lithiasis.

Physical examination was normal, and the only significant finding in serum was a C-reactive protein concentration (PCR) of 12 mg/l (RR: 0–6 mg/l), whereas the remainder of biochemical analytes were within normal range (Table 1A). However, flow cytometry (UF-1000i, Sysmex) and urine strip test (COBAS U411) revealed a pH of 5, with proteinuria, hematuria, and leukocyturia (Table 1B). Urine sediment was composed of a high amount of the flat hexagonal crystals typical of cystine (Figure 1A). In view of these results, a urine HPLC analysis (Biochrom 30Plus Amino Acid Analyzer) was performed at the Department of Metabolic Disorders of the University Hospital of Badajoz, Spain. HPLC demonstrated elevated concentrations of dibasic amino acids (ornithine, lysine, and arginine) and cystine (Table 1B). This finding confirmed the clinical suspicion, although it could not be confirmed either on site by the Brand test or by the simplified method described by our group elsewhere [7] because of a lack of reagents. No crystals were identified on abdominal X-ray, only some phleboliths were identified in the pelvis unrelated to the disease of the patient (Figure 1B).

**Table 1:** Laboratory test results of the patient.

A) Biochemistry		
Parameter	Result	Normal range
Glucose, mg/dL	76	65–110
Urea, mg/dL	43	10–50
Creatinine, mg/dL	1.26	0.60–1.40
Sodium, mmol/L	138	135–153
Potasium, mmol/L	3.9	3.5–5.3
Total bilirubin, mg/dL	1.0	0.1–1.2
LDH, U/L	173	135–225
GPT, U/L	18	5–40
α-Amylase, U/L	44	10–100
PCR, mg/dL	12	0–6

B) Urine test		
Flow cytometry, urine strip test, urine sediment		
Leukocytes, μL	36	0–25
RBCs, μL	582	0–25
Hyaline cylinders, μL	1.1	0.0–3.0
Epithelial cells, μL	5	0–50
Bacteria, μL	22	0–10,000
Proteins, mg	27	0–20
pH	5	
Urine sediment	Abundants cystine crystals	

Amino acid assay		
Arginine, mmol/mol creat.	7.80	1–7
Lysine, mmol/mol creat	156.79	12–52
Cystine, mmol/mol creat.	23.66	1–19
Ornithine, mmol/mol creat.	26.01	0–5

A) Serum biochemistry. B) Flow cytometry, urine test strip, urine sediment, and most significant findings in amino acid tests. Creat, creatinie.

Management of the problem involved hydration and metamizol (575 mg) every 8 h for 3–5 days. The patient was recommended to go to his reference hospital for confirmation of diagnosis, control, and monitoring.

## Discussion

In the light of the findings summarized above and the symptoms described by the patient, a diagnosis was established of non-complicated renal colic secondary to cystinuria. As the patient lived in another autonomous community, once pain was controlled with medication, the patient was discharged with a recommendation to take plenty of liquids and a prescription for analgesics. The patient was advised to go to his reference hospital for confirmation of diagnosis based on an analysis of the composition of calculi (in case of excretion), 24-h determination of urine cystine, characterization of gene mutations, the administration of an adequate treatment, and monitoring. Treatment of cystine calculi by lithotripsy is challenging due to their extreme hardness and high risk of recurrence. A non-invasive therapy is recommended based on hydration, urine alkalinization with potassium citrate or acetazolamide, and the use of chelating agents such as d-penicillamine, tiopronin (α-mercaptopropionylglycine, α-MPG), bucylamine, or captopril to degrade cystine into other soluble compounds that can be easily excreted in urine [8].

Renal colic is a frequent cause of consultation to EDs, especially in summer, and accounts for 90% of cases of full/partial/acute urinary tract obstruction [9]. The signs and symptoms of this disease are those of upper urinary tract obstruction of different etiologies (calculi, clots, to name a few). Therefore, physical examination and differential diagnosis are essential to establishing a final diagnosis, as a wide range of entities can have atypical presentations and supplementary tests including an abdominal radiography and urinary sediment testing in a clinical laboratory can be fundamental. The age of the patient and lack of a personal or family history of interest led to a differential diagnosis focused on urinary tract calculi.

The only remarkable finding on biochemistry and urine tests and the hemogram were increased serum PCR concentrations, high protein concentrations in urine, and hematuria, characteristics of urinary tract calculi. Nevertheless, the conclusive finding was the identification of hexagonal-shaped crystals in urine sediment. The presence of this type of crystals in urine is of clinical significance itself, as they are the manifestation of excess urinary excretion of cystine and are pathognomonic of cystinuria [3]. The amino acid test showed elevated levels of dibasic amino acids and cystine in urine, which confirmed the previous finding. Simple abdominal X-ray did not show any image suggestive of lithiasis, perhaps due to its limited sensitivity to detect cystine calculi [6].

Cystinuria should always be considered in patients with urinary calculi or urinary symptoms, especially if they are young. This problem is associated with a high rate of recurrence and a family history of the disease. As it occurs in other monogenic disorders associated with the formation of calculi, symptoms appear at an early stage and start with the formation of calculi at a young age, occurring very rarely at an advanced age [10]. The risk for calculi in cystinuria throughout life exceeds 50%, and their frequency ranges from a few episodes in the mild type to several episodes per year in the most severe cases. Nevertheless, not all patients with cystinuria develop calculi, as their formation is favored by the presence of factors such as saline balance, the excretion of calculogenesis inhibitors, or environmental factors [11]. We recommend that urine sediment examination is performed in all patients with renal colic, regardless of their age and in the absence of previous episodes. This is a non-invasive, cost-effective test that can lead to findings that are unusual and may remain unnoticed otherwise.

## Conclusions

Although, this case report clearly describes a case of urinary tract calculi, urine sediment examination in the clinical laboratory demonstrated the occurrence of cystinuria evidenced by the appearance of crystals that are pathognomonic of cystinuria at an advanced age and without a previous history. However, this finding should be supported by a confirmatory diagnostic protocol that includes urine amino acid determination, the analysis of calculi, 24-h determination of cystine in urine and a genetic study to characterize genetic mutations in the responsible gene.

This clinical case reveals the important role of urine sediment testing for the correct interpretation and classification of crystals and its use as a diagnostic tool in the clinical laboratory. This study also highlights the need for diagnostic protocols that include routine microscopic urine sediment testing.
